# It’s all in our skin—*Skin autofluorescence*—A promising outcome predictor in cardiac surgery: A single centre cohort study

**DOI:** 10.1371/journal.pone.0234847

**Published:** 2020-06-29

**Authors:** Britt Hofmann, Kristin Anja Gerull, Katja Bloch, Marcus Riemer, Christian Erbs, Anna Fröhlich, Sissy Richter, Martin Ehrhardt, Christopher Zitterbart, Friederike Fee Bartel, Pauline Siegel, Andreas Wienke, Rolf-Edgar Silber, Andreas Simm

**Affiliations:** 1 Department of Cardiac Surgery, Mid-German Heart Center, University Hospital Halle (Saale), Halle, Germany; 2 Department of Gynecology, St. Elisabeth and St. Barbara Hospital Halle (Saale), Halle, Germany; 3 Department of Surgery, Hospital Aarberg, Spital Netz Bern, Aarberg, Switzerland; 4 Institute of Medical Epidemiology, Biostatistics and Informatics, Martin-Luther-University Halle-Wittenberg, Halle (Saale), Germany; IRCCS Policlinico S.Donato, ITALY

## Abstract

**Background:**

The optimum risk score determining perioperative mortality and morbidity in cardiac surgery remains debated. Advanced glycation end products (AGEs) derived from glycaemic and oxidative stress accumulate to a comparable amount in skin and the cardiovascular system leading to a decline in organ function. We aimed to study the association between AGE accumulation measured as skin autofluorescence (sAF) and the outcome of cardiac surgery patients.

**Methods:**

Between April 2008 and November 2016, data from 758 consecutive patients undergoing coronary artery bypass grafting, aortic valve replacement or a combined procedure were analyzed. Skin autofluorescence was measured using an autofluorescence reader. Beside mortality, for the combined categorical morbidity outcome of each patient failure of the cardiac-, pulmonary-, renal- and cerebral system, as well as reoperation and wound healing disorders were counted. Patients without or with only one of the outcomes were assigned zero points whereas more than one outcome failure resulted in one point. Odds ratios (ORs) were estimated in multivariable logistic regression analysis with other preoperative parameters and the established cardiac surgery risk score systems EuroSCORE II and STS score.

**Results:**

Skin autofluorescence as non-invasive marker of tissue glycation provided the best prognostic value in identifying patients with major morbidity risks after cardiac surgery (OR = 3.13; 95%CI 2.16–4.54). With respect to mortality prediction the STS score (OR = 1.24; 95%CI 1.03–1.5) was superior compared to the EuroSCORE II (OR = 1.17: 95%CI 0.96–1.43), but not superior when compared to sAF (OR = 6.04; 95%CI 2.44–14.95).

**Conclusion:**

This finding suggests that skin autofluorescence is a good biomarker candidate to assess the perioperative risk of patients in cardiac surgery. Since the EuroSCORE does not contain a morbidity component, in our view further sAF measurement is an option.

## Introduction

There is still a wide debate concerning the performance of commonly used risk prediction models in assessing the risk of patients undergoing cardiac surgery. Cardiac surgeons are more and more faced with old and geriatric patients and their wish not only to know the predicted surgical mortality but the expected morbidity rate as well. The European System for Cardiac Operative Risk Evaluation II (EuroSCORE II) is one of the most widely used scores to predict perioperative mortality in cardiac surgery, together with The Society of Thoracic Surgeons (STS) score. However, only the STS score also offers morbidity risk prediction. Still, both scores lack of a tailored mortality and morbidity prediction. Inclusion of a specific biomarker may refine these models. Elderly people and patients are becoming more and more heterogeneous. [1]

In many patients chronological age does not match their biological age. Biomarkers reflecting the biological age, thereby predicting impaired responsiveness or poorer outcome better than chronological age, are of great interest. One of these biomarkers are Advanced Glycation End-Products (AGEs). By crosslinking proteins (leading to tissue stiffening) and induction of inflammation, AGEs seem to be involved as a basic mechanism of ageing in the development of degenerative diseases such as diabetes, nephropathy, dementia and cardiovascular disease. [2, 3] AGEs are the irreversible result of the Maillard reaction that starts by a non-enzymatic modification of a protein by sugars. Initially this is a reversible reaction that leads to the formation of unstable compounds, the so-called Schiff base. [3] The latter undergoes molecular rearrangements over a period of days, yielding more stable Amadori products like the haemoglobin A1c (HbA1c). Over weeks and months these turn, through a series of oxidation reactions, into stable compounds known as advanced glycation end products. AGEs are formed in extracellular matrix proteins such as collagen, laminin and elastin, thereby altering the physiological properties of the matrix and increasing its stiffness. [4] Tissues that are rich in extracellular matrix and long-lived proteins such as the vessels, the heart and the skin are affected by AGE modifications, i.e. glycation, glycoxidation and cross-linking. [3, 5] Since specific AGEs exhibit intrinsic fluorescence properties, measuring the skin autofluorescence (sAF) to estimate the AGE tissue burden is now widely established. [6, 7] So far, some studies have provided first evidence, that non-invasive sAF correlates well with cardiac and vascular mortality and morbidity. [2, 7–11] A recently published systematic review summarizing 10 studies on patients with chronic renal disease, peripheral artery disease and diabetes indicate that skin autofluorescence can be considered predictors of mortality in patients at high risk. [12]

This study was designed to compare the mortality and morbidity outcome prediction of the EuroSCORE II, the STS score and the non-invasive skin autofluorescence in patients undergoing elective cardiac surgery.

## Materials and methods

### Preoperative patient characteristics

Between April 2008 and November 2016, data from 758 consecutive patients undergoing elective first-time cardiac surgery (isolated coronary artery bypass grafting, or isolated aortic valve replacement, or coronary artery bypass grafting with aortic valve replacement) were prospectively recorded. Exclusion criteria were age < 18 years, LVEF ≤ 20%, emergency operations, reoperations, procedures with surgery of the mitral valve and aorta, myocardial infarction 24 h before surgery, preoperative liver disease, preoperative dialysis, hematological or oncological systemic disease or systemic infection. The study was approved by the local Ethics Committee of the Medical Faculty of Martin-Luther-University Halle-Wittenberg (ethical approval number 162/03.08.05/3) and was carried out in accordance with the Declaration of Helsinki guidelines. The patients involved in the study gave their written informed consent. At the preoperative visit, all study parameters including skin autofluorescence and blood for laboratory analysis were determined.

### Categorical outcomes

The categorical outcome parameters for this study were orientated on the known major adverse cardiac and cerebrovascular events (MACCE) and Non-MACCE criteria in cardiac surgery [13] and the STS score risk model outcomes. For the combined categorical outcome of each patient, failure of the cardiac- (perioperative myocardial infarction according to the universal definition from 2012 [14], new atrial fibrillation or ventricular fibrillation, or cardiogenic shock), pulmonary- (reintubation, prolonged ventilation > 24 h), renal- (dialysis postoperatively, or serum creatinine ≥ 350 μmol/l, or increase of 3x most recent preoperative creatinine level) and cerebral-system (confirmed neurological deficit > 24 h), as well as reoperation (for bleeding/tamponade, graft occlusion, other cardiac and non-cardiac reasons) and wound healing disorders (deep sternal wound infection) were counted.

Patients without or with only one of the outcomes listed above were assigned zero points whereas more than one outcome failure (OF) was assigned one point. According to this the patient population was divided into two groups (patients with ≤ 1 OF and patients with > 1 OF) according to the presence of postoperative multimorbidity outcome.

Perioperative was defined as period of time extending from cardiac surgery until the patient was discharged. Mortality and morbidity within this time period was recorded for all study patients.

### Skin autofluorescence

SAF was measured using the validated sAF reader (DiagnOptics, Groningen) in arbitrary units (a.u.), as described previously. [2, 6, 11]

### Statistics

Categorical variables were expressed as frequencies and percentages. Metric variables were expressed as mean ± standard deviation (SD). To predict risk factors for morbidity and in-hospital mortality, simple and multivariable logistic regression analyses were done to estimate the odds ratio (OR) with their 95% confidence intervals (CI) and p-values. Discrimination was assessed using the area under the receiver operating characteristic curve (AUROC). AUROC analysis was also utilized to calculate cut off values, sensitivity, specificity, and overall correctness. Finally, cut off points were identified by obtaining the best Youden index.

## Results

### Baseline characteristics

In the present study, we assessed 758 patients (mean age: 67.8 ± 9.1 years, range 29–87 years) with elective first-time isolated coronary artery bypass grafting, or isolated aortic valve replacement, or coronary artery bypass grafting with aortic valve replacement. All patients were operated with extra corporal circulation. Two hundred and thirty-five (31%) patients were females and they were slightly younger (66.9 ± 9 years) than males (69.8 ± 9 years). The pre-operative left ventricular ejection fraction of our patients was normal (55.6 ± 12.5%). Further demographic and clinical data of the study individuals in total and the survivors with low (OF≤1) and high (OF>1) morbidity outcome are shown in Table 1.

**Table 1:** Demographic and clinical characteristics of all patients (n = 758), patients with low (n = 640; 3 deaths) and patients with high postoperative morbidity outcome (n = 118; 13 deaths).

Variable	Total population n = 758	Patients with OF ≤ 1 n = 640	Patients with OF > 1 n = 118	Simple logistic regression		
				OR	95%CI	p-value
Age (years)	67.8 ± 9.1	67.2 ± 9.2	71.3 ± 7.3	1.05	1.03 – 1.08	<0.0001
Weight (kg)	82.9 ± 15.8	82.6 ± 15.6	84.7 ± 16.8	1.01	0.99 – 1.02	0.2
BMI (kg/m^2^)	28.7 ± 4.8	28.6 ± 4.6	29.5 ± 5.5	1.04	0.99 – 1.08	0.06
Gender female (%)	235 (31)	195 (30.5)	40 (33.9)	0.85	0.56 – 1.3	0.46
LVEF, %	55.6 ± 12.5	56.3 ± 12.2	51.5 ± 13.7	0.97	0.96 – 0.99	0.0002
Hypertension, %	95.4	95.3	95.8	0.89	0.64 – 1.25	0.52
Diabetes mellitus (%)	296 (39.1)	238 (37.2)	58 (49.2)	1.42	1.1–1.83	0.009
COPD, %	10.3	9.5	14.4	1.23	0.81–1.87	0.35
ECA, %	26.7	25.8	31.4	1.2	0.89 – 1.63	0.24
CC (ml/min)	85.9 ± 34.7	88.3 ± 34.3	73.2 ± 33.9	0.98	0.98 – 0.99	<0.0001
sAF (AU)	2.8 ± 0.6	2.7 ± 0.6	3.2 ± 0.6	3.73	2.6 – 5.34	<0.0001
EuroSCORE II	2.27 ± 2.01	2.18 ± 1.96	2.78 ± 2.21	1.13	1.04–1.23	0.005
STS-MR	1.67 ± 1.59	1.52 ± 1.32	2.45 ± 2.46	1.33	1.19–1.49	<0.0001
STS-MMR	13.27 ± 6.93	12.59 ± 6.21	16.97 ± 9.25	1.08	1.05 – 1.11	<0.0001
Operation type						
Isolated CABG (%)	508 (67)	432 (67.5)	76 (64.4)	0.87	0.58 – 1.31	0.51
Isolated AVR (%)	142 (18.7)	121 (18.9)	21 (17.8)	0.92	0.56–1.55	0.78
CABG + AVR (%)	108 (14.3)	87 (13.6)	21 (17.8)	1.38	0.82–2.32	0.24
Operation times						
Total surgical procedure (min)	188.0 ± 54.2	183.9 ± 48.5	210.5 ± 74.6	1.01	1.004–1.011	<0.0001
Cardiopulmonary bypass (min)	87.1 ± 39.9	84.6 ± 35.1	100.4 ± 58	1.01	1.004–1.015	0.0005
Aortic cross-clamp time (min)	61.6 ± 31.2	51.7 ± 25.8	61.8 ± 37.6	1.01	1.005–1.018	0.0008

Data are presented as mean ± standard deviation (SD) or percent (%). AVR, aortic valve replacement; BMI, body mass index; CC, Cockroft-Gault creatinine clearance; ECA, extracardiac arteriopathy; n, number of cases; LVEF, left ventricular ejection fraction; OF, organ failure; sAF, skin autofluorescence; STS-MR, STS mortality risk; STS-MMR, STS morbidity/mortality risk.

Patients without postoperative organ failure were n = 348 (1 dead), one organ problem according to MACCE-, Non-MACCE criteria and the STS score risk model outcomes occurred in 292 patients (2 dead). High morbidity outcome defined as two or more organ problems was noticed in 118 patients (2 OF n = 80, 2 deceased; 3 OF n = 28, 5 deceased; 4 OF n = 5, 1 deceased; 5 OF n = 5, all deceased). The mean length of hospital stay in the present study was 15.26 ± 8 days (range from 2–107 days) and in-hospital mortality was 2.1% (n = 16). The causes of postoperative organ failure are shown in Table 2.

**Table 2:** Causes of postoperative organ failure.

causes of organ failure (OF)		OF 1 (n = 292)	OF 2 (n = 80)	OF 3 (n = 28)	OF 4 (n = 5)	OF 5 (n = 5)
**cardiac**	new atrial fibrillation	168	44	14	3	1
	new pacemaker	9	1	1	0	2
	myocardial infarction	7	4	3	0	3
	low cardiac output syndrome	1	5	4	2	5
**pulmonary**	prolonged ventilation	12	14	16	3	4
	pneumonia	6	11	8	1	1
**renal**	dialysis	12	19	11	4	5
	creatinine increase	4	6	0	0	0
**cerebral**	postoperative delirium	46	38	11	2	3
	stroke (CT confirmed)	6	3	3	1	0
**reoperation**		4	3	10	3	0
**wound infection**	deep sternal	17	12	3	1	1

### Independent predictors of morbidity and mortality

Multivariable logistic regression analysis revealed age, lower left ventricular ejection fraction and sAF, reflecting accumulation of late glycation products, as independent factors associated with higher morbidity (Table 3).

**Table 3:** Multivariable analysis for demographic and clinical variables predicting morbidity.

Variables	Multivariable Logistic Regression		
	OR	95%CI	p-value
Age	1.03	1.003–1.066	0.048
LVEF	0.98	0.96–0.99	0.006
Diabetes mellitus	1.2	0.91–1.58	0.2
Creatinine clearance	0.99	0.99–1.003	0.19
sAF	2.8	1.92–4.14	< 0.0001

OR: Odds Ratio; 95%-CI: 95%- confidence interval for OR; LVEF, left ventricular ejection fraction; sAF, skin autofluorescence.

Only sAF was also an independent predictor for in-hospital mortality (OR = 5.66; 95% CI 2.19–14.6; p = 0.0003). With respect to the established scores, the STS score morbidity/mortality risk and sAF were independent predictors of postoperative morbidity (Table 4), supporting the predictive value of sAF in this context. Further, we assessed sAF and STS mortality/morbidity risk for goodness-of-fit with the Hosmer-Lemeshow test (p = 0.31). The discriminative abilities in terms of high in-hospital morbidity are provided in Table 5.

**Table 4:** Multivariable analysis for scores and markers predicting morbidity.

Variables	Multivariable Logistic Regression		
	OR	95%CI	p-value
STS MMR	1.06	1.03 – 1.09	0.0001
sAF	3.13	2.16–4.54	< 0.0001

OR: Odds Ratio; 95%-CI: 95%- confidence interval for OR; sAF, skin autofluorescence; STS-MMR, STS score morbidity/mortality risk.

**Table 5:** Comparison of discriminative and predictive abilities for in-hospital morbidity.

	Discrimination					
	AUROC ± SE		95%CI		p-value	
STS MMR	0.66 ± 0.03		0.62 – 0.69		< 0.0001	
Age	0.63 ± 0.03		0.59–0.69		< 0.0001	
LVEF	0.61 ± 0.03		0.57–0.64		0.0001	
sAF	0.70 ± 0.03		0.67–0.73		< 0.0001	
**Predictive Factors**	***Cutoff Point***	***Youden Index***	***Sensitivity (%)***	***Specificity (%)***		***Overall Correctness (%)***
STS MMR	> 13.99*	0.25	55.9	69.5		62.7
Age	> 64*	0.19	83.1	35.9		59.5
LVEF	≤ 58*	0.19	66.9	52.7		59.8
sAF	> 2.82*	0.31	74.6	56.6		65.6

AUROC: area under the receiver operating characteristic curve; CI: confidence interval; LVEF, left ventricular ejection fraction; sAF, skin autofluorescence; STS-MMR, STS score morbidity/mortality risk.

*Value giving the best Youden index.

**Fig 1:**
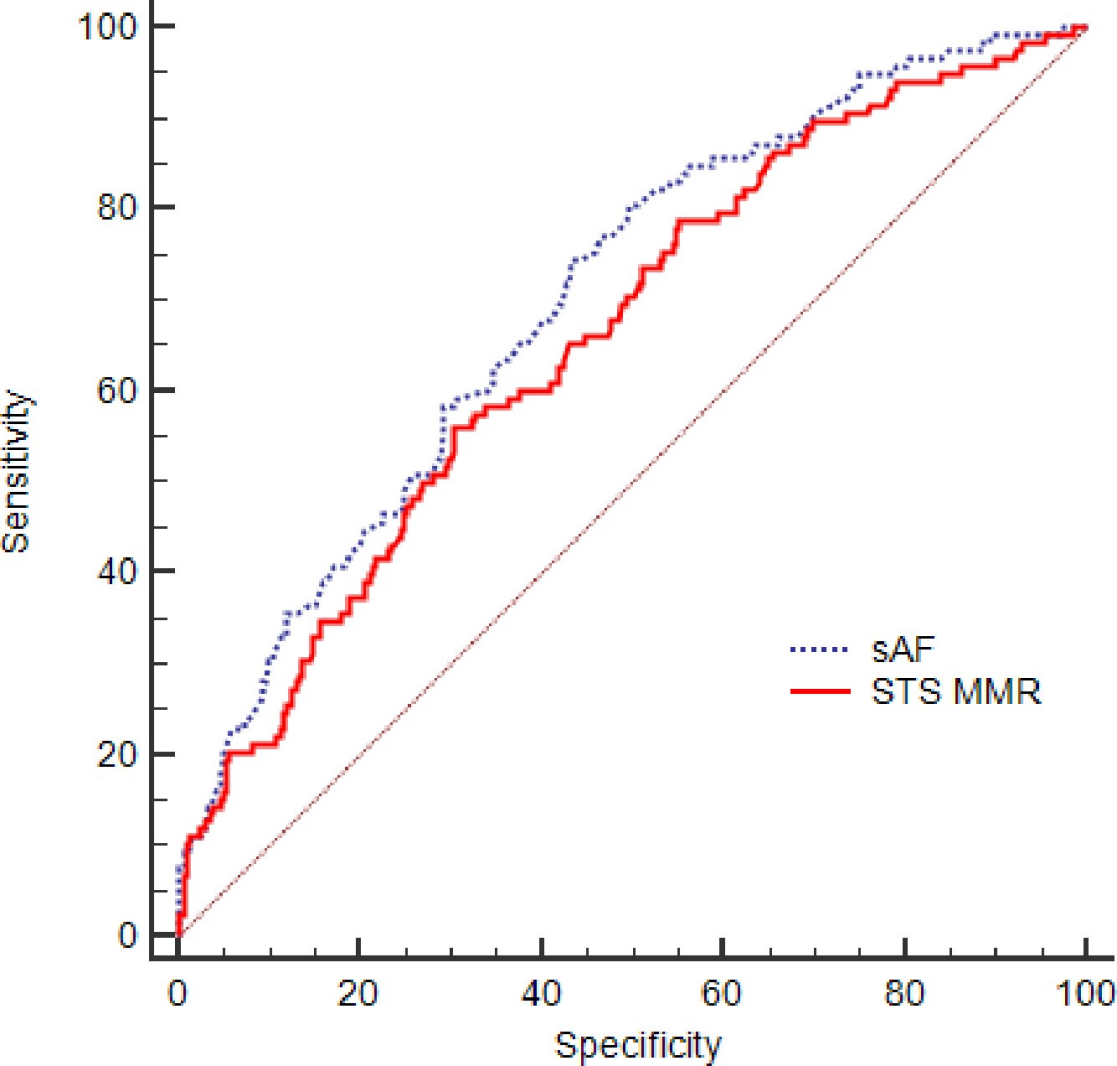
Receiver operating characteristic (ROC) curves of the measured skin autofluorescence (sAF) and the calculated STS morbidity/mortality risk (STS MMR) in the whole patient population with respect to the outcome high morbidity (OF>1).

AUROC analysis confirms that the discriminative power of sAF is comparable with the STS mortality/morbidity risk results. To assess the predictive value of each parameter for in-hospital morbidity we determined sensitivity, specificity, and overall correctness of prediction. SAF and STS morbidity/mortality risk had the better Youden index and higher overall prediction correctness. The discrimination results (ROC test) favored the sAF (Fig 1, Table 5).

**Fig 2:**
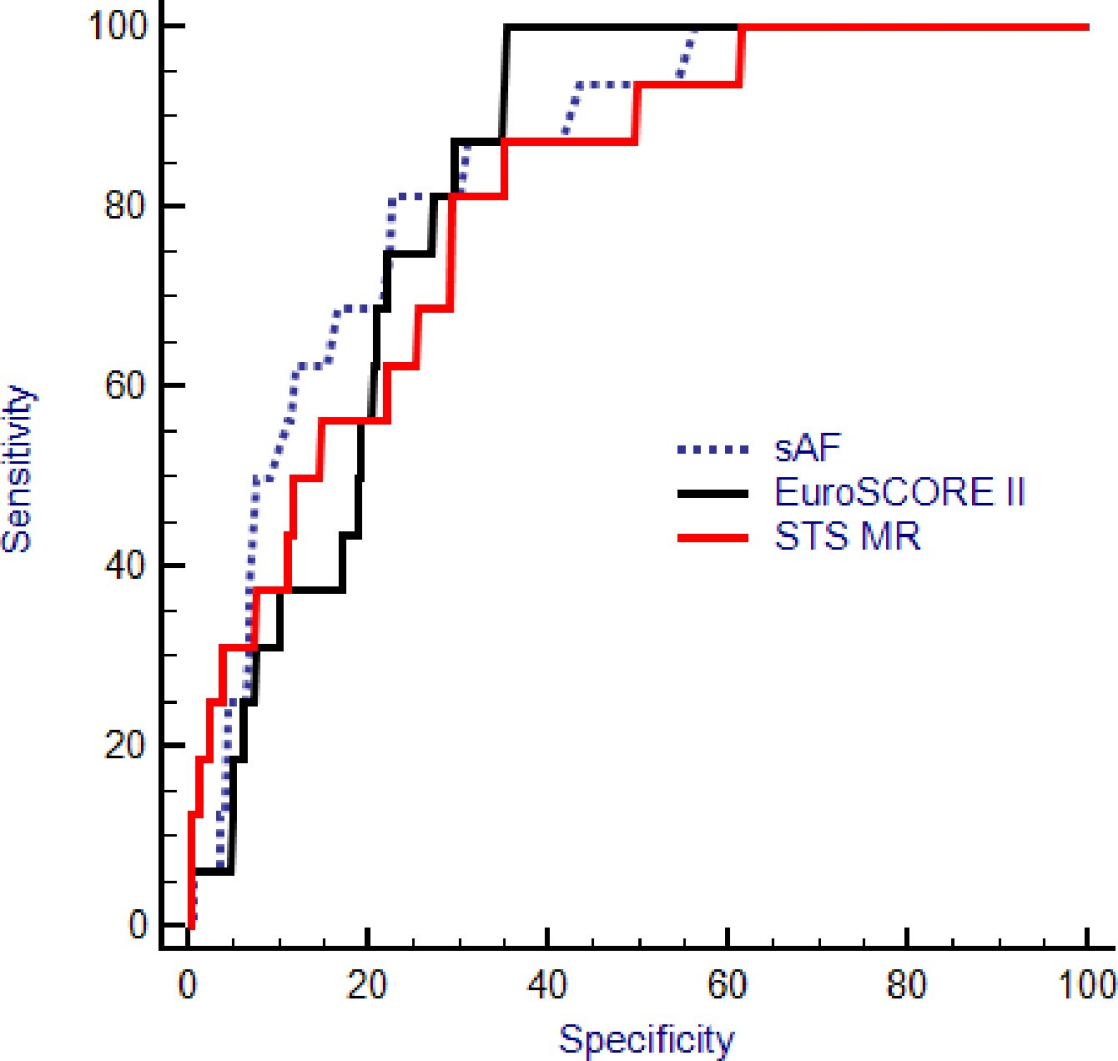
Receiver operating characteristic (ROC) curves of the measured skin autofluorescence (sAF) and the calculated STS mortality risk (STS MR) and EuroSCORE II in the whole patient population with respect to the outcome mortality.

With respect to mortality prediction the STS score (OR = 1.24; 95%CI 1.03–1.5; p = 0.02) was superior in mortality risk prediction compared to the EuroSCORE II (OR = 1.17; 95%CI 0.96–1.43; p = 0.12), but not superior when compared to sAF (OR = 6.04; 95%CI 2.44–14.95, p = 0.0001). However, sAF and EuroSCORE II had the better Youden index and the higher overall prediction correctness (Fig 2, Table 6).

**Table 6:** Comparison of discriminative and predictive abilities for in-hospital mortality.

	Discrimination					
	AUROC ± SE		95%CI		p-value	
STS MR	0.81 ± 0.05		0.78 – 0.84		< 0.0001	
EuroSCORE II	0.82 ± 0.03		0.79–0.85		< 0.0001	
sAF	0.84 ± 0.04		0.81–0.87		< 0.0001	
**Predictive Factors**	***Cutoff Point***	***Youden Index***	***Sensitivity (%)***	***Specificity (%)***		***Overall Correctness (%)***
STS MR	> 1.62*	0.52	87.5	64.7		76.1
EuroSCORE II	> 2.08*	0.64	100	64.4		82.2
sAF	> 3.22*	0.58	81.2	77.2		79.2

AUROC: area under the receiver operating characteristic curve; CI: confidence interval; sAF, skin autofluorescence; STS-MR, STS score mortality risk.

*Value giving the best Youden index.

## Discussion

Most of the clinical outcome studies in cardiac surgery concentrate on mortality as the central outcome. The STS score is the only scoring system in cardiac surgery also predicting a postoperative morbidity risk. This study combined the analysis of a biomarker of ageing in patients and the morbidity outcome after cardiac surgery. The most important findings of this study are that the non-invasive parameter sAF is an independent and predictive biomarker of postoperative morbidity and mortality in cardiac surgery comparable to the well-known risk-scores.

Ageing is marked by an accumulation of tissue damage, for example by increased amounts of oxidized and glycated proteins. These changes in the vessels and the myocardium are at least in part responsible for organ dysfunction or a reduced reserve in response to a challenge e.g. an operation. Due to demographic changes and improved medical treatment, the number of older patients undergoing highly invasive procedures such as complex cardiac surgery is increasing. Age is a major risk factor for postoperative mortality [15], we also saw a correlation in our study using categorical parameters for morbidity as the endpoint. On the other hand, the odds ratio is low, which can be due to the increasing heterogeneity with age [1]. To overcome the discrepancy between the chronological and the biological age, biomarkers reflecting the biological age of a person were identified and became of great interest. One of these biomarkers, which potentially can be used in a clinical situation, is accumulation of AGEs. Increasing evidence indicate that AGEs negatively influence the function of the heart and the vascular system and are able to predict patients at risk for cardiovascular disease [16]. AGE-associated skin autofluorescence was used to assess the prognosis of already diseased humans [17]. As mentioned before [2], the generation of AGEs especially in the extracellular matrix is more pronounced during maintained hyperglycaemia [18], oxidative and carbonyl stress [19]. AGE modified amino acids are cleared from the blood by glomerular filtration. However, a certain amount accumulates over a lifetime in proteins with a low turnover [20]. Our previous data indicate that this is mainly based on the highly modified and cross-linked collagen fractions [2]. The accumulation of AGEs in the extracellular matrix of the vessels and the heart causes functional alterations leading to structural changes in collagen [21], endothelial dysfunction and the synthesis of proinflammatory cytokines [22]. Recent data from Raposeiras-Roubin et al. showed a predictive value of AGEs for the development of post-infarction heart failure [17]. In line with our data [2, 11], den Dekker et al. [23] reported that AGEs reflected by sAF are associated with the burden of atherosclerosis. A recently published systematic review summarizing 10 studies on patients with chronic renal disease, peripheral artery disease and diabetes indicate that skin autofluorescence is a predictor of mortality in patients at high risk [12]. Taking all these considerations into account, accumulation of AGEs seem to be a good biomarker candidate to assess the perioperative risk of patients in cardiac surgery. Since the EuroSCORE does not contain a morbidity component, in our view further sAF measurement proves out. Overall, the SAF is consistently better as a predictive parameter for postoperative outcome and morbidity, but not more elaborate in its investigation than the established scores.

To our knowledge this is the first study demonstrating that the non-invasively measured skin autofluorescence is an independent and predictive biomarker for the outcome after cardiac surgery.

Some limitations of the study should be noted. Our study population was a population with a low to medium risk profile. This results in small numbers of events in terms of mortality and morbidity implying limited statistical power. SAF measurement is limited by the fact that not all AGEs exhibit fluorescent properties. The study results are based on logistic regression analysis with hospital mortality as yes/no outcome because the timing of death during such a short period as hospital stay is negligible. Therefore, logistic regression was preferred instead of Cox regression.

Despite the above, we believe that our data reflect the pathophysiological and clinical situation of our patients and open the field to study the non-invasive biomarker skin autofluorescence.

## Supporting information

S1 Table Clinical data set. (XLSX)

## Abbreviations and acronyms

AGEs: advanced glycation end products
a.u.: arbitrary unit
EuroSCORE II: European System for Cardiac Operative Risk Evaluation II
MACCE: major adverse cardiac and cerebrovascular events
sAF: skin autofluorescence
STS score: Society of Thoracic Surgeons score
